# General practitioners' opinions on how to improve treatment of mental disorders in primary health care. Interviews with one hundred Norwegian general practitioners

**DOI:** 10.1186/1472-6963-10-35

**Published:** 2010-02-09

**Authors:** Arnstein Mykletun, Ann Kristin Knudsen, Tone Tangen, Simon Øverland

**Affiliations:** 1 Research Section for Mental Health Epidemiology, Research Centre for Health Promotion, Faculty of Psychology, University of Bergen, Bergen, Norway; 2 Division of Mental Health, Norwegian Institute of Public Health, Oslo, Norway; 3 Institute of Clinical Medicine, University of Bergen, Bergen, Norway

## Abstract

**Background:**

Improvements in treatment of mental disorders are repeatedly called for. General practitioners (GPs) are responsible for the majority of treatment of mental disorders. Consequently, we interviewed GPs about their opinions on how treatment of mental disorders in primary health care contexts could be improved.

**Methods:**

Among GPs affiliated within the Norwegian reimbursement system, we approached 353, and made contact with 246 GP's. One-hundred of these agreed to participate in our study, and 95 of them expressed opinions on how to improve treatment of mental disorders. The telephone interviews were based on open-ended questions, responses were transcribed continuously, and content analysis was applied. Results are presented both as frequency tables of common responses, and as qualitative descriptions and quotations of opinions.

**Results:**

Nearly all (95%) of the GPs had suggestions on how to improve treatment of mental disorders in primary health care. Increased capacity in secondary health care was suggested by 59% of GPs. Suggestions of improved collaboration with secondary health care were also common (57%), as were improvements of GPs' skills and knowledge relevant for diagnosing and treating mental disorders (40%) and more time for patients with mental disorders in GP contexts (40%).

**Conclusions:**

The GPs' suggestions are in line with international research and debate. It is thought-provoking that the majority of GPs call for increased capacity in secondary care, and also better collaboration with secondary care. Some GPs made comparisons to the health care system for physical disorders, which is described as better-functioning. Our study identified no simple short-term cost-effective interventions likely to improve treatment for mental disorders within primary health care. Under-treatment of mental disorders is, however, also associated with significant financial burdens.

## Background

Most patients who suffer from severe mental disorders are treated in specialized health care services [1]. Such conditions are however far less frequent than common mental disorders, like anxiety and depression, which increasingly are described as major public health challenges [2,3]. In the present health care system, most patients with a common mental disorder are offered treatment in a general practice setting. In Norway, this is organized within a listed patient system.

Many experts claim that depression is easy to diagnose and treat [4]. Yet, several studies have demonstrated that depression is subject to under-treatment [2,5]. From an Australian study, it is estimated that less than 30% of patients with depression receive proper treatment [6], while the corresponding figure for asthma is around 90% [7]. In Norway, results from the Nord-Trøndelag Health Survey (HUNT) revealed that only a minority of those with high levels of anxiety and depression symptoms have ever received treatment for this [8]. Indication of under-treatment is also found among patients who are awarded a disability pension for a mental disorder [9], and among adolescents with anxiety and depression [10].

Results from the largest epidemiologic study of changes in prevalence and treatment of mental disorders, the US based National Comorbidity Survey [11], does not suggest any change in prevalence from 1990 to 2003. However, there are clear indications of a considerable increase in treatment of mental disorders during this period. The increased treatment rate has largely occurred in primary health care settings and in the form of prescriptions of antidepressants and SSRI's. The paradox is that most of the increase in prescription is for patients who do not fulfil the diagnostic criteria for mental disorders, while many with a diagnosable disorder still receive no treatment. It is therefore argued that this costly increased use of medication has not had any notable impact on the prevalence of mental disorders in the general population [11]. A recent meta-analysis of antidepressants efficacy based on US Food and Drug Administration (FDA) approval data suggested that SSRI's were effective in severe depression, but had no better effect than placebo for less severe and minor depressive conditions [12], which are the most frequently seen in primary care settings.

There is increasing consensus that common mental disorders have serious consequences. The WHO World Health Survey (WHS) concluded that depression gives stronger decrements in perceived functional status than angina, arthritis, asthma and diabetes [13]. This conclusion is in line with results from the first WHO-initiated Global Burden of Disease study [3]. Norwegian longitudinal studies have suggested that depression increases mortality in general, across the specific causes of death. The odds ratios for risk for mortality from depression were of comparable magnitude with those from smoking [14,15]. Anxiety and depression are also strong and independent risk factors for disability pension, also for disability pensions awarded for somatic conditions [16]. Hence common mental disorders and their consequences constitute a major public health challenge.

There has been a strong focus on diagnosing and treatment of common mental disorders in primary health care [17-19]. In the UK, a new system for primary mental health care based on evidence-based psychological treatments is now being developed [2]. In Norway, there have been calls for improvements in treatment of people with mental disorders, both in primary and secondary care. The aim of the present study was to assess whether primary health care physicians believe there is a need for improved treatment of mental disorders in primary health care, and if so, which actions they think should be taken to meet those needs.

## Methods

Our sample of informants was recruited from all general practitioners (GPs) in Norway enrolled in the public reimbursement system. The vast majority of GPs are enrolled in this system, as the market for private healthcare without public reimbursement is very limited in Norway. To get a random sample of GPs, we first selected a letter for each Norwegian county. Then, we listed all GPs within each county with a surname starting with the given letter. From this list of 353 GPs, we contacted potential participants by telephone until we reached the pre-defined sample size of 100. The participants were called in a pre-defined sequence by quotas making sure that the county-wise distribution of GPs reflected the population size of each county. We were unable to retrieve a correct phone number for 40 of GPs. We further failed to get in touch with 67 of them after having attempted five times, 87 were not interested in participating, and 59 did not return the calls later despite agreeing to do this. A total of 906 calls were made. We offered no financial incentive for participating in the study.

The data presented in this paper are based on the telephone interviews with the 100 general practitioners within primary health care who agreed to participate in the study as informants. Thirty percent of the informants were female, and the average number of years as a primary care practitioner was 17.8 (range 0.5 - 35.0). This distribution is in accordance with another study which has used Norwegian GPs as informants, where it was concluded that the distribution was representative for Norwegian GPs in general [20]. The data collection was financed by the Norwegian Directorate of Health, and the researchers were funded by the Norwegian Institute of Public Health and the Norwegian Research Counsel.

Two trained interviewers were instructed to initiate every interview with presenting their errand on behalf of the Norwegian Directorate of Health, and to arrange with the GP's secretaries when the interviews with the GP could take place. The interviews were carried out in the periods between 10.12.07 to 20.12.07, and 03.01.08 to 18.01.08, and lasted between four and 20 minutes.

Each GP was given the following background information at the beginning of the interview: "We call you on behalf of the Norwegian Directorate of Health. You are randomly drawn from the list of GPs in Norway enrolled in the reimbursement system, and we call you to collect opinions, thoughts and experiences relevant for improving mental health care in primary care. The information collected will be used to inform the directorate in their continuous process of improving mental health services in GP settings, and will also be published as a research report in a medical journal. We will ensure anonymity of every informant in the study. Are you willing to participate in this study?" A note of the oral informed consent was made, and the interview then started. All the informants who participated were offered copies of the report given to the Norwegian Directorate of Health as a reimbursement for their contribution. This was sent to the GPs at the time of presenting the results to the directorate (February 2008).

The interview commenced with the opening question: "Can anything be done to improve treatment of mental disorders in primary health care?" If the GP stated that no such improvements could be done (n = 5) the interview ended there. The remaining GPs (n = 95) were asked the follow-up question:*"What can be done?"*. The question was asked open ended to avoid leading the answers in any particular direction. The replies (in total 307) were transcribed during the interviews, and content analysis was applied to categorise and quantify the responses [21,22]. This approach was the best one available combining the two following needs: We wanted to approach the GPs with open-ended questions to avoid directing their attention in any direction as a response to specific questions asked. Further, we needed to be able to quantify responses as to the content of the GPs advice, reflections and opinions.

Categories were made after all interviews were transcribed and on the basis of reading the responses. Two of the authors (AM and AKK) suggested categories independently and compared the categories afterwards. Themes were derived from the data on two levels as outlined in Table 1. The unit of analysis was defined according to content, so that the content always should be possible to place in one category only. This resulted in some units consisting of one sentence only, others of two or more sentences. The coding was performed by one of the authors (AKK), and another author (AM) coded some of the sentences for reliability with results compared with very good agreement. The categories applied are presented in Table 1 with four main domains, and further specification of two of the categories.

**Table 1:** "What can be done to improve treatment of mental disorders in primary health care?"

	%	95% confidence interval
*Answers with a focus on secondary health care (all together)*	*82%*	*74 - 90*

Need for improved capacity in specialized health care	59%	49 - 69

Need for improved co-operation between primary and secondary health care, and other public services	57%	47 - 67

Need for quality improvements in secondary health care services	12%	5 - 18

*Answers with a focus on primary health care (all together)*	*63%*	*54 - 73*

Need for increased knowledge and competence among the general practitioners	40%	30 - 50

Need for extended time when treating patients with mental disorder	40%	30 - 50

Need to change structures or content of the treatment in primary health care	13%	6 - 19

*Answers with a focus on societal interventions*	*11%*	*4 - 17*

*Answers with a focus on patient characteristics*	*5%*	*1 - 10*

Coded answers based on transcribed interviews with the general practitioners.

Cohen's Kappa Coefficient was applied to test the reliability of the categorisation [23]. A research technician was given a minimal instruction (15 minutes) on how to code the comments into categories. Agreement between the original coding (performed by AKK) and the second coding was Kappa 0.91 for the four domains (as specified in Table 1), and 0.73 for all categories combined. This is regarded as *very good *and *good *agreement, respectively [23].

The presentation of the results will give the proportion of replies in each category, with typical examples of the replies within each category. No more than one quotation from each participant will be referenced in the text.

The project was evaluated by the regional committee for ethics in medical research, which had no objections to the study.

## Results

Of the 100 GPs interviewed, five did not suggest that something could be done to improve treatment of mental disorders in primary health care. The remaining 95 were asked what could be done (Table 1). The majority of the GPs suggestions emphasized needs for improvement in secondary health care (82% (95% CI 74 - 90)), specifically in terms of need for increased capacity (59% (95% CI 49 - 69)) and better collaboration between the health services (57% (95% CI 47 - 67)). Two out of three GPs did also address the need for improvement in primary health care (63% (95% CI 54 - 73)), in particular need for increased knowledge and competence among general practitioners regarding these disorders (40% (95% CI 30 - 50)) and need for extended time limits when treating these patients (40% (95% CI 30 - 50)). In the following, each of these main categories of replies is presented with typical quotations.

### Increased capacity in secondary care (59%)

Statements in this category included difficulty with gaining contact and access in secondary health care, long waiting-lists, not enough psychologists and psychiatrists and experiences of unevenly distribution of these across the country and between urban and rural areas. A typical quote in this category was simply *"There is a need for increased capacity in secondary health care"*. Other examples of statements were: "*It is hard to refer patients [to specialised health services] when one does not know where to look for them, and when those you try to refer the patient to does not reply to mail nor calls"*, *"We need shorter waiting lists in secondary health care. It is not good enough when people have to remain on a waiting list three months before treatment is offered." *and "... *where I work it is far too hard to get access for your patients"*. Many suggested an increase in numbers of psychiatrists and psychologists, and easier access to these: *"In this area we have a need for more psychologists. Psychologists should be distributed where they are needed" *and *". it may seem like those [psychiatrists and psychologists] that runs private clinics have a high selection of whom they choose to treat as patients. They should receive more new patients and finish more of the cases they already have"*. Some addressed possibilities for increased capacity through improved treatment routines in secondary health care:*"I think clinical psychologists are exempt from the daily trivialities. They let their patients receive treatment for a long time and often exclusively for specific diagnoses. They should to a larger degree become part of the public health care system. Treatments should be shortened to 20-30 sessions followed by an evaluation of status. They should also be forced to see more patients in acute phases. A limited number of sessions per patient could help more to get started. It is too easy for psychologists to refrain from accepting patients. Better to provide some help for many than much help for only a few." *Some also asked for low threshold services in secondary health care: "*Increased contact with psychiatric nurses would give a low threshold offer for referral"*.

### Need for improved co-operation between primary and secondary health care, and other public services (57%)

In contrast to statements regarding easier access to secondary health care in general, which were coded in the above mentioned category, statements in this category address the reciprocal co-operation between the two levels of health care. The following quote serve as an example: *"It should be possible to refer to specialised health care for a quick consultation (as is possible with somatic conditions) about diagnosis and possible treatments, and then I could perform the treatment myself in cases where I am capable." *A simpler and less formal access to counselling and supervision was also asked for: *" [We need] improved access to supervision from psychologists or psychiatrists in secondary health care on our treatment of these patients.", "I have a psychologist in the office next door. We have interdisciplinary co-operation. This works well"*. Enhanced quality on case summaries and quicker notification when the patient is in treatment was also mentioned *" .little information is given to the GP when the patient has been admitted to a hospital" *and *"faster feedback like epicrises [.] when hospitals and similar institutions have been in contact with the patient"*.

### More knowledge and competence in general practice (40%)

Several of the GPs addressed a need for more knowledge and competence among the GPs regarding diagnosing and treatment. Some offered general statements like: *"Need for more knowledge among general practitioners on mental disorders"*, but many offered specific comments: *"There should be mandatory training for general practitioners. Being left to random representatives from pharmaceutical companies or random courses is not sufficient." *And: *" [...] psychiatry could become integrated in the house office training to provide experience before we start working." *Some also asked for specific competence in psychological therapies: *"This [course in cognitive therapy] should be part of the physician's supplementary education and not just a voluntary selected course"*.

### Extended time for consultations (40%)

Many underlined the need for extended consultation time for patients with mental disorders in general practice: *"Mental disorders demand more time for treatment and consultation than other patients." "Time-constraints, we have a limited amount of time available for each patient and that especially goes for these patients." *Suggestions for changes in the reimbursement system occurred: *"Better time can be achieved through changing the reimbursement system. As of today, we are rewarded for doing short consultations with many patients. This is not optimal for patients with mental disorders, who need more time." "Differentiated time rates, special rates for treating mental disorders [...]. In reality, the physicians lose money treating mental disorders since the time spent could be used treating other conditions. A salary based on use of time could increase the physician's motivation for doing these consultations thoroughly."*

### Other answers

Twelve percent (95% CI 5 - 18) of the GPs emphasized a need for quality-improvement in secondary health care, like in the following quote *"Where I work there is a lack of qualified professionals in secondary health care"*. Relatively few (11%, 95% CI: 4 -17) asked for societal interventions: *"Information [.] through media about mental disorders is important"*, and some few were concerned about patient characteristics (5%, 95% CI: 1 - 10) that mattered in treatment: *"Some patients have some requirements for treatment that might not be possible to fulfil, and experience that it is not good enough for them being treated by a general practitioner" *(Table 1).

## Discussion

General practitioners do most of the treatment of common mental disorders, however, a high rate among them (59%) asks for increased capacity in secondary health care for further referral of this patient group. This might reflect that many general practitioners feel inadequate facing patients with mental disorders, and experiences little support and nowhere else to get help for these patients. In Norway, sales figures for SSRIs doubled from 1989 to 1999, and have since increased by another 10-15% annually [24], most of it due to increased prescription in general practice [11]. This is in line with current treatment guidelines for depression, recommending two trials with antidepressant medication before referring the patient to specialised health services [25]. In spite of this, the general practitioners clearly expressed wish for increased possibilities for referral may indicate that medication is perceived as an insufficient tool when treating common mental disorders like depression.

If a substantially larger proportion of the patients presenting symptoms of anxiety and depression in general practice should be referred to specialized health care, the current system would collapse. In the UK, an alternative approach to this public health challenge is being tried out: In the project Improved Access to Psychological Therapies (IAPT) psychological treatment centres are established across the country to deliver low threshold evidence-based psychological short-term therapies. It is planned as a primary health care service in the sense that patients may self-refer and as an alternative to general practitioner administered medication-based treatments. The general practitioners are also free to refer suitable patients to the centres [2,26]. In its full scale, it is assumed that a total of 10,000 new therapists are needed to run this system throughout the UK. Having received some specific training, these therapists are supposed to work independently, but under close supervision by psychologists and psychiatrists [2]. To date, there are no published evaluations of the project or its pilots, but the experiences made will be relevant also outside UK borders.

Many of the general practitioners (57%) reported a need for improved co-operation with specialized health care services, both in terms of easier access for further referrals of patients, and availability of guidance and supervision. Some of the physicians compared the mental health care system with how health care for somatic health problems works, emphasizing the differences in how easy it is to get further examinations and specialist evaluations for somatic health problems compared to mental health problems. Some described that they would be happy to carry out the actual treatment themselves if they could get some initial help from a psychologist or psychiatrist in making a diagnosis and develop a plan for treatment. It seems a core problem that the specialists, representing the highest level of competence on mental disorders, are largely found to be unavailable for those who treat most of the patients in this domain.

A model where the practitioner do patient consultations under supervision from a specialist has been tried out in international studies, resulting in improved treatment outcomes [27], but also higher costs [18,27]. Joint consultation between physicians and psychiatrists has also been tried out in a Norwegian setting, with positive results: A one hour joint consultation was described as useful for giving the right diagnosis and plan a good treatment schedule for the patient [28].

Many of the general practitioners expressed a need for more knowledge and competence regarding mental disorders (40%), both in terms of using the correct diagnostic label and to offer efficient treatment. Under-recognition of mental disorder in general practice is previously described [13,17,29], and is completely in line with the general practitioners own descriptions regarding this issue.

One obvious response to this challenge would be to increase training and courses in mental health for general practitioners. This has been attempted previously: In a highly cited study, general practitioners in the UK were randomized to an educational program aiming to increase their sensitivity and specificity in recognition of depression, while the control group did not receive the program [17]. Despite that the participants themselves reported the program as being well suited and that it had improved their skills as intended, no difference between the groups in terms of recognition of depression or improvement in depression scores among the patients with known depression was found after six months [17]. A systematic review concluded in line with this: There is a considerable scope for improving recognition and treatment of depression in general practice, but frequently employed strategies for doing so, like treatment guidelines and educational programs, are most often not effective [27]. An improved co-operation between primary and secondary health care, with continuous updating and guidance, is found to be a more effective strategy [27], but also more expensive [18].

40% of the practitioners suggested prolonged consultation time for patients with mental disorders. Some stated that this was a question about the physicians own priorities, while others emphasized a need for revising the reimbursement system the physicians work within. Many argued that the current reimbursement system fails to provide an economic incentive to allocate sufficient time for these patients. The changes the physicians ask for would inflict large costs and this must be a subject of cost-effectiveness evaluations before any implementation.

### Strengths and weaknesses of the study

The primary strength of our study is the use of open-ended questions about how treatment of mental disorders in primary health care can be improved, reducing demand characteristics through leading predefined answers. The study is large enough to provide reasonable margins around the proportions of answers given. Our county-level stratified random sampling should to some extent ensure that the sample was representative of Norwegian general practitioners, as also indicated through the reasonable distribution of gender and years of practice [20]. However, only 100 of the 353 selected, and 247 identified GPs agreed to attend, and we cannot exclude the possibility that non-informants would have different opinions and views, potentially biasing our results. The response rate achieved is thus a challenge. The good reliability given in Kappa Coefficient of the coding is also a strength of this study. A complete list of all answers will be provided upon contacting the corresponding author if so desired.

## Conclusion

Norwegian general practitioners suggest four main interventions to improve treatment of mental disorders in primary health care: Increase the capacity in secondary health care; improve co-operation between primary and secondary health care; increase the knowledge about mental disorders in primary health care; and allow more time to treat patients with mental disorders. Many of these suggestions are in line with recommendations based on international research. The study did not help to identify any available interventions that in short time would imply improvements. Effective interventions will be expensive, but so is mental disorders left untreated.

## Abbreviations

GP: general practitioner; CI: confidence interval; HUNT: The Nord-Trøndelag Health Survey; FDA: Food and Drug Administration; WHO: World Health Organization; UK: United Kingdom; SSRI: Selective serotonin reuptake inhibitors; IAPT: Improved Access to Psychological Therapies.

## Competing interests

The authors declare that they have no competing interests.

## Authors' contributions

AM contributed to the conception and design of the study, raising funding, in the analysis and interpretation of data, and in drafting and revising of the manuscript. AKK contributed to the design of the study, in the data collection, in analysis and interpretation of data and in revising of the manuscript. TT contributed to the analysing and interpretation of data, and in drafting and revising of the manuscript. SØ contributed to the conception and design of the study, in the analysis and interpretation of data, and in drafting and revising of the manuscript. All authors have read and approved the final manuscript.

## Pre-publication history

The pre-publication history for this paper can be accessed here:

http://www.biomedcentral.com/1472-6963/10/35/prepub
